# Quantification of myocardial late gadolinium enhancement using synthetic inversion recovery imaging

**DOI:** 10.1186/1532-429X-17-S1-O8

**Published:** 2015-02-03

**Authors:** Akos Varga-Szemes, Rob J van der Geest, Bruce S Spottiswoode, Giuseppe Muscogiuri, Carlo N De Cecco, Pal Suranyi, Wolfgang G Rehwald, Joseph U Schoepf

**Affiliations:** 1 Department of Radiology and Radiological Science, Medical University of South Carolina, Charleston, SC, USA; 2 Leiden University Medical Center, Leiden, Netherlands; 3 Siemens Medical Solutions, Chicago, IL, USA; 4 University of Rome "Sapienza", Rome, Italy; 5 Duke Cardiovasular Magnetic Resonance Center, Durham, NC, USA

## Background

Inversion recovery (IR) late gadolinium enhancement (LGE) cardiac magnetic resonance (CMR) is generally used for post-contrast infarct detection in the myocardium. Novel technological advancements allow for the generation of synthetic IR images based on the pixel-by-pixel T1 values. Theoretically, synthetic IR images can be retrospectively generated at any inversion time (TI). In this study, we aimed to evaluate the diagnostic value of synthetic phase-sensitive IR (PSIR) images for the quantification of myocardial LGE.

## Methods

The Institutional Review Board approved the study protocol. Consecutive patients (n=31) underwent CMR using 1.5T Siemens MAGNETOM Avanto (Siemens AG, Erlangen, Germany) equipment. Conventional PSIR acquisition and fast T1-mapping (investigational prototype modified look-locker IR, MOLLI, 4(1)3(1)2 sampling scheme) of the heart were performed. This scheme acquires 9 images in 11 heartbeats with 3 inversions. For the MOLLI acquisition, an initial TI of 110ms was used with a 80ms TI increment. Synthetic PSIR images were calculated using an in-house developed, Microsoft Excel based application allowing the generation of these images at any TI. LGE was quantified by two independent readers employing a binary thresholding method using Research MASS analytical software. The accuracy of infarct quantification with the synthetic technique was compared to that of the conventional technique.

## Results

**Figure 1:**
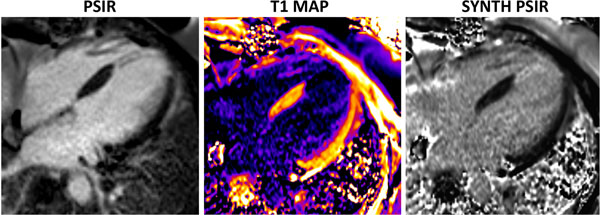


LGE was observed in 11 (35.4%) patients. LGE pattern was consistent with myocardial infarction in all cases. Representative conventional PSIR, as well as corresponding T1 map and synthetic PSIR images are shown in Figure 1. The infarct fraction measured by conventional and synthetic PSIR techniques was 16.5±7.4 and 17.2±7.9%, respectively, and statistically identical (p=0.238). Inter-observer studies revealed excellent agreement between the two readers (Kappa >0.81). There was excellent agreement in the detection of myocardial infarction between the two techniques.

## Conclusions

In this project we have shown the feasibility of LGE detection and quantification using synthetic PSIR images. Synthetic images provide the same accuracy as the conventional PSIR images used in clinical practice as standard of care. With the increasing acceptance and availability of T1 mapping, the need for conventional PSIR images could be omitted in the future. Synthetic PSIR techniques would eliminate the need to optimize the LGE acquisition (TI scout, TI readjustment) without additional scanning time.

## Funding

N/A.

